# Human Health Risk of Ingested Nanoparticles That Are Added as Multifunctional Agents to Paints: an *In Vitro* Study

**DOI:** 10.1371/journal.pone.0083215

**Published:** 2013-12-16

**Authors:** Jean-Pierre Kaiser, Matthias Roesslein, Liliane Diener, Peter Wick

**Affiliations:** Materials-Biology Interactions Laboratory, Empa, Swiss Federal Laboratories for Materials Science and Technology, St. Gallen, Switzerland

## Abstract

Microorganisms growing on painted surfaces are not only an aesthetic problem, but also actively contribute to the weathering and deterioration of materials. A widely used strategy to combat microbial colonization is the addition of biocides to the paint. However, ecotoxic, non-degradable biocides with a broad protection range are now prohibited in Europe, so the paint industry is considering engineered nanoparticles (ENPs) as an alternative biocide. There is concern that ENPs in paint might be released in run-off water and subsequently consumed by animals and/or humans, potentially coming into contact with cells of the gastrointestinal tract and affecting the immune system. Therefore, in the present study we evaluated the cytotoxic effects of three ENPs (nanosilver, nanotitanium dioxide and nanosilicon dioxide) that have a realistic potential for use in paints in the near future. When exposed to nanotitanium dioxide and nanosilicon dioxide in concentrations up to 243 µg/mL for 48 h, neither the gastrointestinal cells (CaCo-2) nor immune system cells (Jurkat) were significantly affected. However, when exposed to nanosilver, several cell parameters were affected, but far less than by silver ions used as a control. No differences in cytotoxicity were observed when cells were exposed to ENP-containing paint particles, compared with the same paint particles without ENPs. Paint particles containing ENPs did not affect cell morphology, the release of reactive oxygen species or cytokines, cell activity or cell death in a different manner to the same paint particles without ENPs. The results suggest that paints doped with ENPs do not pose an additional acute health hazard for humans.

## Introduction

Painted surfaces contain biodegradable organic compounds that can be used as nutrients by various types of microorganisms. Such microbial colonization has a major effect on the weathering and deterioration of materials [1,2] and so biocides are added to protect the paint. Organic-based, biodegradable biocides are only active for a limited time and are unable to protect surfaces that are exposed to wet conditions for several years. Ecotoxic, non-degradable biocides with a broad range of protection have been abolished because the *European Biocidal Products Directive 98/8/EC* (BPD) requires an environmental risk assessment for biocidal products prior to their introduction on the European market [3].

The current idea is to replace organic-based biodegradable biocides with engineered nanoparticles (ENPs). ENPs can be fixed more easily in the paint matrix, which reduces the risk of leaching into the surrounding environment, compared with conventional water-soluble biocides [4]. ENPs in paints not only have biocidal effects, they also improve paint properties, such as water repellence, scratch resistance and increased hydrophobicity [5]. Thus, the paint industry is considering using ENPs in surface coatings as a biocide (nanosilver), UV-light absorber/biocide (nanotitanium dioxide) or as a hardener (nanosilicon dioxide). If the paint and lacquer industry succeeds in producing ENP-doped paint formulations with improved properties, it might become one of the biggest end-user of ENPs such as nanosilver and nanotitanium dioxide [6].

A concern is that incorporation of ENPs in paints might result in greater release of ENPs into the environment with subsequent health effects. ENPs released by abrasion or weathering might find their way into the environment and be inhaled or ingested. The lung is the most sensitive port of entry for ENP uptake [7]. Despite the lungs’ natural defence systems, some ENPs might persist and induce symptoms of stress, inflammation or have more severe health effects. Therefore, the effects of ENPs on the different cells in the lung have been intensively studied. Because ENPs differ in terms of their structural characteristics (morphology, size, shape and length), surface properties (surface chemistry, surface charge) and chemical composition, they have different cytotoxic effects. However, it has been demonstrated that most of the inhaled ENPs are discharged from the lung by the respiratory mucociliary escalator [8]. Ultrafine particles are also taken up by alveolar macrophages and similarly discharged. The ENPs finally end up in the gastrointestinal tract and are excreted [9].

ENPs incorporated in paints may also be released from painted facades during rain and accumulate in the surface water. The surface water might be consumed by animals or humans and by that the ENPs are ingested and will come in contact with epithelial cells of the gastrointestinal tract (CaCo-2 cells). Further it cannot be excluded that the ENPs find their way into open wounds and by that these ENPs might affect our immune systems. Therefore we selected for the evaluation of adverse effects on the gastrointestinal tract cells and on cells of the immune system a simplified *in vitro* model with epithelial cells of the gastrointestinal tract (CaCo-2) and cells of the immune system (Jurkat).

Nanosilver, nanotitanium dioxide and nanosilicon dioxide have a realistic potential to be used in the near future as paint additives. Therefore in the present study our aim was to evaluate if the use of ENP-doped paints increases the acute health risk, with a special focus on ingestion. We investigated in this study possible cytotoxic effects of nanosilver, nanotitanium dioxide and nanosilicon dioxide, as well as the cytotoxicity of the corresponding paints (with and without ENPs) on CaCo-2 cells (epithelial cells of the human gastrointestinal tract) and Jurkat cells (immune-responsive cells). The selected *in vitro* cytotoxicity parameters will provide a good indication, if the investigated particles might cause additional acute health risks.

The cytotoxicity tests comprised five different parameters that are commonly used in the acute toxicology assessments of ENPs [10]. The following parameters were investigated in this cytotoxicity study: cell morphology, particle uptake, release of reactive oxygen species, cell activity and cell viability.

To simulate natural conditions, conventional paints with and without ENPs were altered by exposure to UV light prior to analysis of their cytotoxic potential. Because the amount of released ENPs would also depend on the mechanical stress applied, cells were exposed to a broad range of concentrations (up to 243 µg/mL) to include the scenario of accidentally high exposure.

## Materials and Methods

### Cell cultures

CaCo-2 cells were obtained from Health Protection Agency Culture Collections (Salisbury, UK: order no. 86010202). The cells were cultured in minimum essential medium Eagle (Sigma, Buchs, Switzerland), with 10% heat-inactivated fetal calf serum (FCS) (Life Technologies, Basel, Switzerland), 1% penicillin–streptomycin–neomycin–solution (PSN; Life Technologies), 1% glutamine solution (Life Technologies), 1% non-essential amino acid solution (Sigma) and 1 mmol sodium pyruvate (Life Technologies) under cell culture conditions (5% CO_2_, 95% air and 37°C). The cultures were passaged once weekly.

Jurkat-neo cells are human leukaemic T-lymphocytes (Jurkat cells transfected with a control neo construct; gift from Dr S. J. Korsmeyer, Harvard Medical School, Boston, MA, USA) [11]. Jurkat cells (ATCC, CRL-2898) were cultured in RPMI 1640 medium (Sigma) with 10% heat-inactivated FCS and 1% PSN solution (Life Technologies) under cell culture conditions (5% CO_2_, 95% air and 37°C). The cultures were passaged once weekly.

### Characterization of ENPs and paint particles

ENPs characterization was performed using different physicochemical methods, such as dynamic light scattering, zeta potential analysis and transmission electron microscopy (TEM). The characterization data of the ENPs and paint particles are summarized in Tables 1 and 2, respectively.

**Table 1:** Characteristics of three ENPs examined by different physical-chemical methods, such as dynamic light scattering, zeta potential analysis and transmission electron microscopy (TEM), according to Smulders et al. [12].

	**Nanosilver**	**Nanotitanium dioxide**	**Nanosilicon dioxide**
**Function**	Biocide	Self-cleaning agent	Scratch resistance
**Endotoxin**	No	No	No
**TEM size (nm)**	25 (spherical) to 80–90 (rods)	15	19
**Hydrodynamic diameter (nm)**	91	386	195
**Zeta potential (mV)**	−42	−25	−14
**Shape**	Spheres and rods	Spherical	Spherical

**Table 2:** Paint composition and ENP concentrations.

**Paint sample**	**Matrix**	**Doped with**	**ENP concentration**	**Special property**
Ag-1	Styrene acrylic copolymer	Nanosilver	0.1%	−
Ag-2	Styrene acrylic copolymer	No	−	Control paint without nanosilver
Ti-1	Styrene acrylic copolymer and polysiloxan resin	Nanotitanium dioxide	4.3%	With microtitanium dioxide (19.4%)
Ti-2	Styrene acrylic copolymer and polysiloxan resin	No	−	Control paint without nano-, but with microtitanium dioxide (20.3%)
Ti-3	Styrene acrylic copolymer and polysiloxan resin	No	−	Control paint without nano- and microtitanium dioxides
Si-1	Styrene acrylic copolymer	Nanosilicon dioxide	5%	−
Si-2	Styrene acrylic copolymer	No	−	Control paint without nanosilicon dioxide

As described by Smulders et al. [12], the presence of endotoxin was analysed with a kinetic chromogenic limulus amebocyte lysate assay (Charles Rivers Laboratories, Sulzfeld, Germany). The detection range was 0.005–50 EU/mL according to the manufacturer.

Changes in the size (i.e. agglomeration) of nanosilver and nanotitanium dioxide during incubation in culture media were analysed by a particle tracking method with a NanoSight LM20 instrument. The particles (nanosilver and nanotitanium dioxide, 1 mg/mL) were incubated in culture media with 10% FCS at 37°C for 0, 3, 6, 12, 18, 24 and 48 h. The samples were diluted 10-fold prior to measurement, which resulted in a final concentration of 100 µg/mL.

### Ageing and milling of the paints

The paints were applied with and without ENPs onto fibrocement panels that had been preconditioned according to EN1062-11:2002 (3 cycles of 24 h in water at 23 ± 2°C and 24 h drying in an oven at 50 ± 2°C) prior to application of the paints at a concentration of 0.3–0.4 kg/m^2^ in two layers without primer. The panels were then conditioned for 7 days in a climatic chamber at 23 ± 2°C and 50% relative humidity before exposure to UV light (UVB, 313 nm) in an accelerated weathering chamber for 500 h using the test method UNI EN10686:1998 (63 cycles with lamps switched on for 4 h at 60 ± 2°C and 4 h with lamps switched off and water in condensation at 50 ± 2°C). The altered paints were removed from the panels using a Traber rotary platform abrader (Traber Industries, North Tonawanda, NY, USA) according to the standard ISO7784-2:2006; (load 500 g, 500 cycles). Milling of the abraded, altered paints occurred in two stages. First, milling was performed using a Fritsch Mortar Grinder (Mill Pulverisette, Type 06002-866, Fritsch, Idar-Oberstein, Germany). The milling medium, made of zirconia, was conditioned by using 70% EtOH/H_2_O (v/v) solution. The mass charge ratio used for milling was 1 : 2 (4 balls of 20 g and 160 g of paint powder). The grinding time was 40 min and the rotor speed was 320 rpm. Second, the paint particles were remilled using a PM 100 Planetary Ball Mill, Retsch Grinding Jar (Retsch, Haan, Germany). Sapphire (α-Al_2_O_3_) balls of 20 mm diameter for dry milling were used until a homogeneous population of paint particles was obtained.

### Determination of proteins adsorbed on ENPs

Nanosilver and nanotitanium dioxide (1 mg/mL) were added to human blood serum (Sigma) and sonicated for 5 min, followed by incubation at 37°C for 3, 6, 12, 24 and 48 h. Negative controls (nanosilver and nanotitanium dioxide, 1 mg/mL) were incubated in distilled water and processed the same way. Human blood serum was used as a positive control. The ENPs were centrifuged (25,000*g*, 5 min) after incubation and washed 5 times with phosphate-buffered saline. SDS-loading buffer (100 µL) was added to the remaining pellet, which comprised ENPs with adsorbed peptides/proteins. The samples were cooked for 5 min at 95°C prior to separation of the proteins by sodium dodecylsulfate polyacrylamide gel electrophoresis. Detached and separated proteins were visualized with a Dodeca Silver Stain Kit (Bio-Rad, Reinach, Switzerland).

### *In vitro* analysis

Cell morphology was analysed after exposure of CaCo-2 and Jurkat cells to (1) paint particles Ag-1, Ag-2, nanosilver and ionic silver; (2) paint particles Ti-1, Ti-2, Ti-3 and nanotitanium dioxide; and (3) paint particles Si-1 and Si-2 and nanosilicon dioxide, up to concentrations of 243 µg/mL for 24 and 48 h. Cell morphology was analysed by bright-field microscopy using a phase contrast method at ×100 (Nikon-Diaphot, Egg, CH).

TEM of incorporated particles was performed with a Zeiss 900 TEM (Carl Zeiss MicroImaging, Jena, Germany) at 80 kV, described in detail by Thurnherr et al. [11].

The release of reactive oxygen species (ROS) was estimated with the reactive dye 2’,7’-dichlorodihydrofluorescein-diacetate (H_2_DCF-DA), as described by Kaiser et al. [13]. 3-Morpholinosydnonimine hydrochloride (1 mM) was used as positive control.

The cytokine assays (IL-8 ELISA Ready-SET-Go and IL-2 ELISA Ready-SET-Go) were performed according to the manufacturer’s protocol (eBioscience, Frankfurt, Germany). Tumour necrosis factor alpha (50 ng/mL; eBioscience) was used in positive control cultures (CaCo-2) to induce the release of interleukin 8 (IL-8). Phorbol-12-myristate-13-acetate (1 µg/mL; Sigma) and phytohaemaglutin (5 µg/mL; Sigma) were used in positive control cultures (Jurkat) to induce the release of IL-2.

Cell activity was analysed according the manufacturer’s protocol (Cell Titer 96, Promega, Wallisellen, Switzerland). Cadmium chloride (25 µM) was used as positive control.

Apoptosis/necrosis in cell cultures was quantitatively analysed by flow cytometric analysis based on the binding of annexin V fluorescein to phosphatidyl serine and incorporation of propidium iodide to distinguish between apoptotic and late apoptotic/necrotic cells. The procedure is described in detail by Kaiser et al. [13]. Cadmium chloride (25 µM) was used as positive control.

### Statistical analysis

The experiments were repeated independently three times. Significant effects were determined using Student’s t-test. Differences were considered significant at p < 0.05. In the Figures, data are presented as the mean of three independent experiments and the standard error of the mean (SEM) over the mean experimental values of each of the three independent experiments.

## Results

### Characteristics of ENPs and paint particles

The characteristics of the different ENPs have been extensively analysed [12] and additional parameters were investigated in the present study (i.e. NanoSight, SDS-PAGE). The data are summarized in Table 1. In addition, changes in the agglomeration state of nanosilver and nanotitanium dioxide were analysed after incubating the particles in media with 10% FCS over 48 h. The size distribution of nanosilver was in the range of 25–300 nm, with maxima of approximately 125 nm. Immediately the silver nanoparticles contacted the culture media, they formed agglomerates that were very stable as evidenced by the lack of visible changes of the agglomerated nanosilver particles during the 48-h incubation (Figure S1A).

Nanotitanium dioxide behaved differently. Its size distribution remained in the range of 5–150 nm, indicating a stabilizing effect of serum proteins. A large amount of non-agglomerated nanotitanium dioxide was observed after dispersing the particles in the culture medium. However, with increasing incubation time in the culture medium, nanotitanium dioxide started to form larger agglomerates and the amount of non-agglomerated particles decreased with increasing incubation time. Agglomerates up to 250–300 nm formed during the 48-h incubation period in medium containing FCS (Figure S1B). The agglomeration stages of nanosilver and nanotitanium dioxide are summarized in Table S1.

### Serum protein binding on ENPs

Besides their agglomeration behaviour, the adsorption of peptides and proteins from human blood serum onto nanosilver and nanotitanium dioxide was investigated. Peptides and proteins up to a molecular weight of 250 kilodaltons were immediately bound by the ENPs on contact. The amount of peptides/proteins adsorbed by nanosilver and nanotitanium dioxide particles increased with prolonged incubation and the pattern of peptide/protein adsorption did not alter after subsequent incubation in human blood serum. No remarkable differences were observed between nanosilver and nanotitanium dioxide particles in the binding pattern of adsorbed peptides/proteins.

### Acute toxicity of the ENPs alone in comparison with paint particles with and without the ENPs

#### Cell morphology

The effects on cell morphology of nanosilver, nanotitanium dioxide and nanosilicon dioxide, as well as the corresponding paint particles with and without the ENPs, were investigated. No visible morphological changes of either CaCo-2 or Jurkat cells were observed when cells were exposed to paint particles Ag-1, Ag-2, Ti-1, Ti-2, Ti-3, Si-1 and Si-2 and nanosilicon dioxide up to a concentration of 243 µg/mL for 48 h. Bright-field microscopy showed that cells exposed to nanotitanium dioxide did not change their morphology (Figure S2).

**Figure 1:**
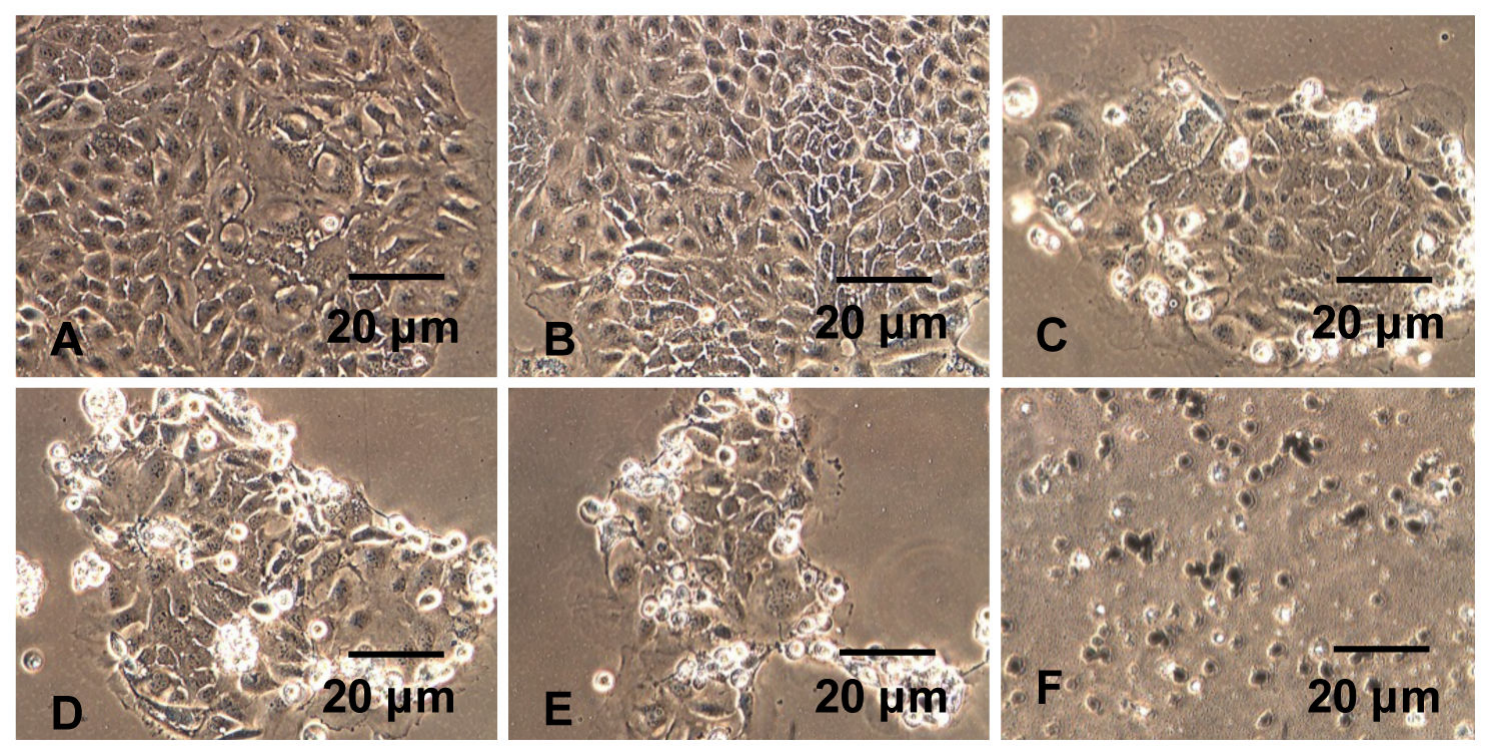
Morphology of gastrointestinal cells (CaCo-2) after exposure to different concentrations of nanosilver for 48 h. A = 1 µg/mL, B = 3 µg/mL, C = 9 µg/mL, D = 27 µg/mL, E = 81 µg/mL, F = 243 µg/mL.

Cells grown in the presence of nanosilver behaved differently. At higher concentrations, it affected cell morphology. CaCo-2 cells at the periphery of cell clusters started to die when exposed to 9 µg/mL nanosilver for 48 h (Figure 1). Jurkat cells were more robust. Only when exposed to 81 µg/mL nanosilver was cell death observed within 48 h and morphological changes.

To discriminate between silver nanoparticles and ionic silver, an additional control (silver sulfate) was introduced. The morphology of the CaCo-2 and Jurkat cells was affected at much lower concentrations compared with nanosilver. CaCo-2 cells exposed to 3 µg/mL ionic silver died within 48 h and the cell clusters disintegrated. Jurkat cells grown in the presence of 1 µg/mL ionic silver showed altered morphology after 48 h of exposure.

#### Cellular uptake

Cellular uptake of the ENPs and the corresponding paint particles (with and without ENPs) was also analysed by TEM. CaCo-2 and Jurkat cells exposed to nanosilver and nanotitanium dioxide incorporated both types of ENP. Nanosilicon dioxide was well suspended and did not agglomerate. Nanosilicon dioxide could not be clearly identified in the cells and therefore it is unclear if it was incorporated into the cells. No notable morphological changes could be observed when cells were exposed to nanosilicon dioxide for 48 h. These observations are in line with the findings of other investigations [14]. The hazard profile of amorphous silicon dioxide is considered to be low. 

**Figure 2:**
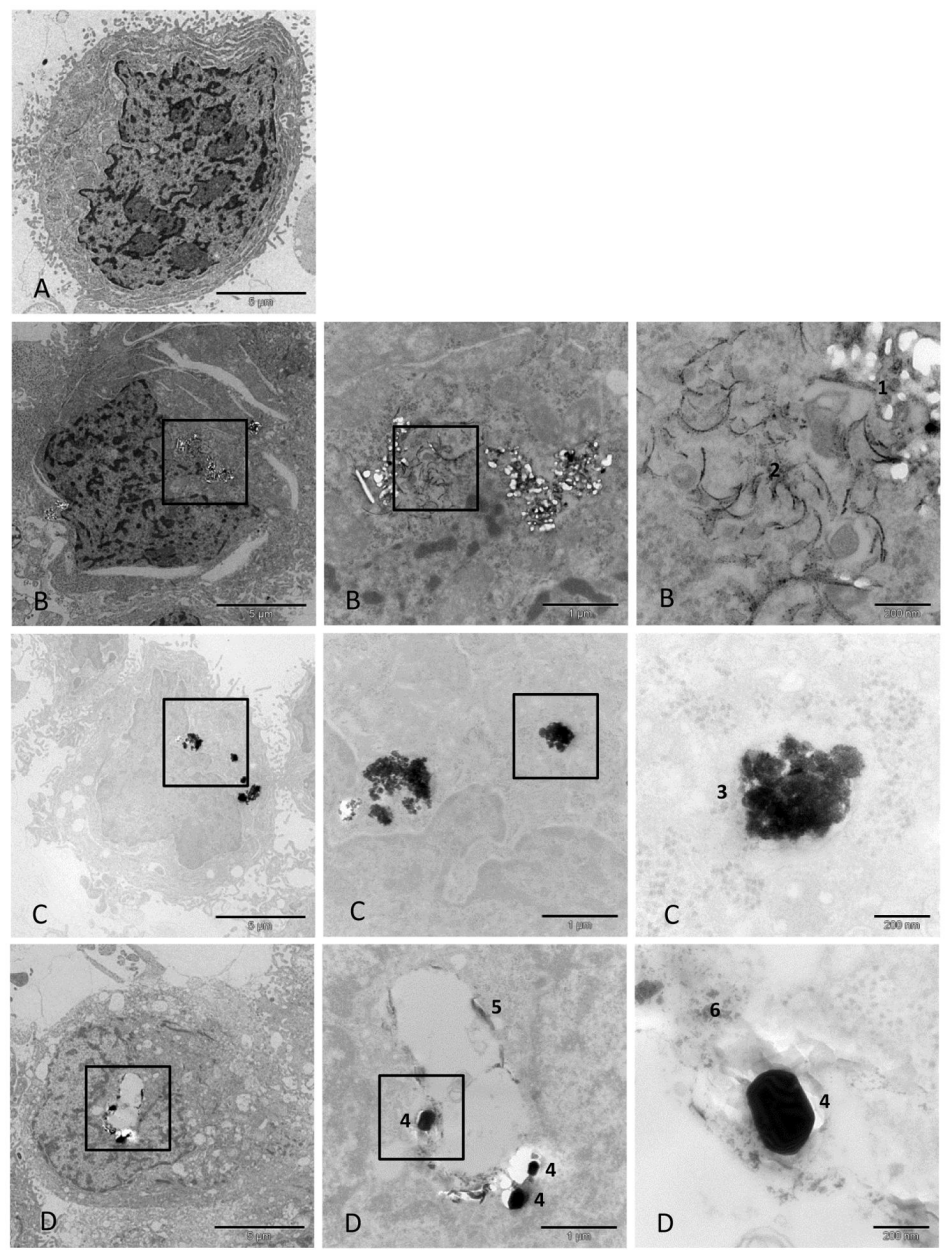
Uptake of nanosilver, nanotitanium dioxide, and paint Ti-1 particles by gastrointestinal cells (CaCo-2). A = Control culture; CaCo-2 cells grown in the absence of ENPs and paint particles. B = CaCo-2 cells exposed to nanosilver (27 µg/mL) for 48 h. A higher amount of nanosilver was incorporated into the cells. Occasionally, the incorporated nanosilver particles formed spherical agglomerates arranged in a row. 1: Nanosilver agglomerates, 2: Nanosilver agglomerates forming a chain. C = CaCo-2 cells exposed to nanotitanium dioxide (243 µg/mL) for 48 h. A higher amount of nanotitanium dioxide was incorporated into the cells. No cytotoxic effects could be observed. 3: Nanotitanium dioxide agglomerates. D = CaCo-2 cells exposed to paint Ti-1 particles show incorporation of the particles. 4: Microtitanium dioxide agglomerates, 5: Agglomerates of paint particles, 6: Nanotitanium dioxide agglomerates.

When CaCo-2 and Jurkat cells were exposed to 27 µg/mL nanosilver or 243 µg/mL nanotitanium dioxide for 48 h, there was an uptake of ENPs (Figures 2A–C and S3A–C). When both cell types were exposed to the titanium dioxide-containing paint particles Ti-1, Ti-2, and Ti-3 in an incubation period of 48 h, nanotitanium dioxide and microtitanum dioxide particles incorporated paint particles were observed to be incorporated (Figures 2D and S3D). The incorporated particles did not affect cell morphology. The data showed that the uptake of smaller paint particles is independent of the presence of ENPs. All three types of paint particles (Ti-1, Ti-2 and Ti-3) were found in the exposed cells.

#### Oxidative stress

**Figure 3:**
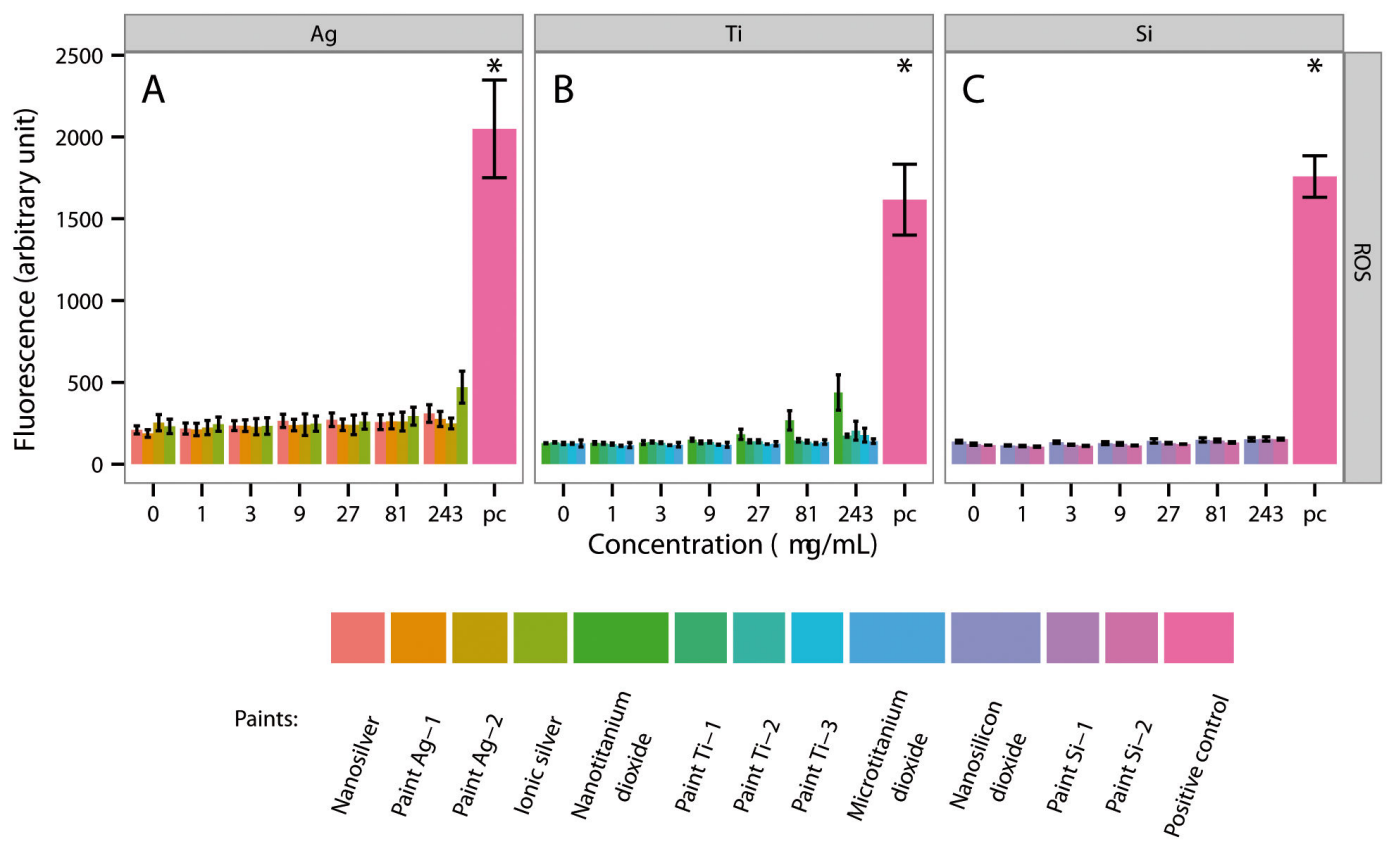
Production of reactive oxygen species (ROS) by gastrointestinal tract cells (CaCo-2) after exposure to ENPs, ionic silver, microtitanium dioxide and aged paint particles for 4 h. A = CaCo-2 cells were exposed to different concentrations of nanosilver, paint Ag-1, Ag-2 and ionic silver. B = CaCo-2 cells were exposed to different concentrations of nanotitanium dioxide, paint Ti-1, Ti-2, Ti-3 and microtitanium dioxide. C = CaCo-2 cells were exposed to different concentrations of nanosilicon dioxide, paint Si-1 and Si-2. * = Significantly different from the control.

When CaCo-2 cells were exposed to nanosilver, ionic silver, nano- or microtitanium dioxide and nanosilicon dioxide, as well as to paint particles Ag-1, Ag-2, Ti-1, Ti-2, Ti-3, Si-1 and Si-2 (243 µg/mL) for 4 h, no significant amounts of ROS were released. Jurkat cells reacted similar. Jurkat cells exposed to nanosilver, ionic silver, nano- or microtitanium dioxide and nanosilicon dioxide, as well as to paint particles Ag-1, Ag-2, Ti-1, Ti-2, Ti-3, Si-1 and Si-2 (243 µg/mL) for 4 h released no significant amounts of ROS. The data on the release of ROS in the presence of the ENPs, ionic silver, microtitanium dioxide and the paint particles are summarized in Figures 3 and S4.

#### Immune response

When CaCo-2 cells were exposed to nanotitanium dioxide and nanosilicon dioxide, respectively, a very low amount of IL-8 was released. However, when the cells were exposed to 27 µg/mL nanosilver for 48 h, higher concentrations of IL-8 were detected. Jurkat cells behaved differently. When exposed to paint particles Ag-1, Ag-2, nanosilver and ionic silver, paint particles Ti-1, Ti-2, Ti-3, nanotitanium dioxide and paint particles Si-1 and Si-2, Jurkat cells released only minor amounts of IL-2.

#### Cell activity and cell viability

**Figure 4:**
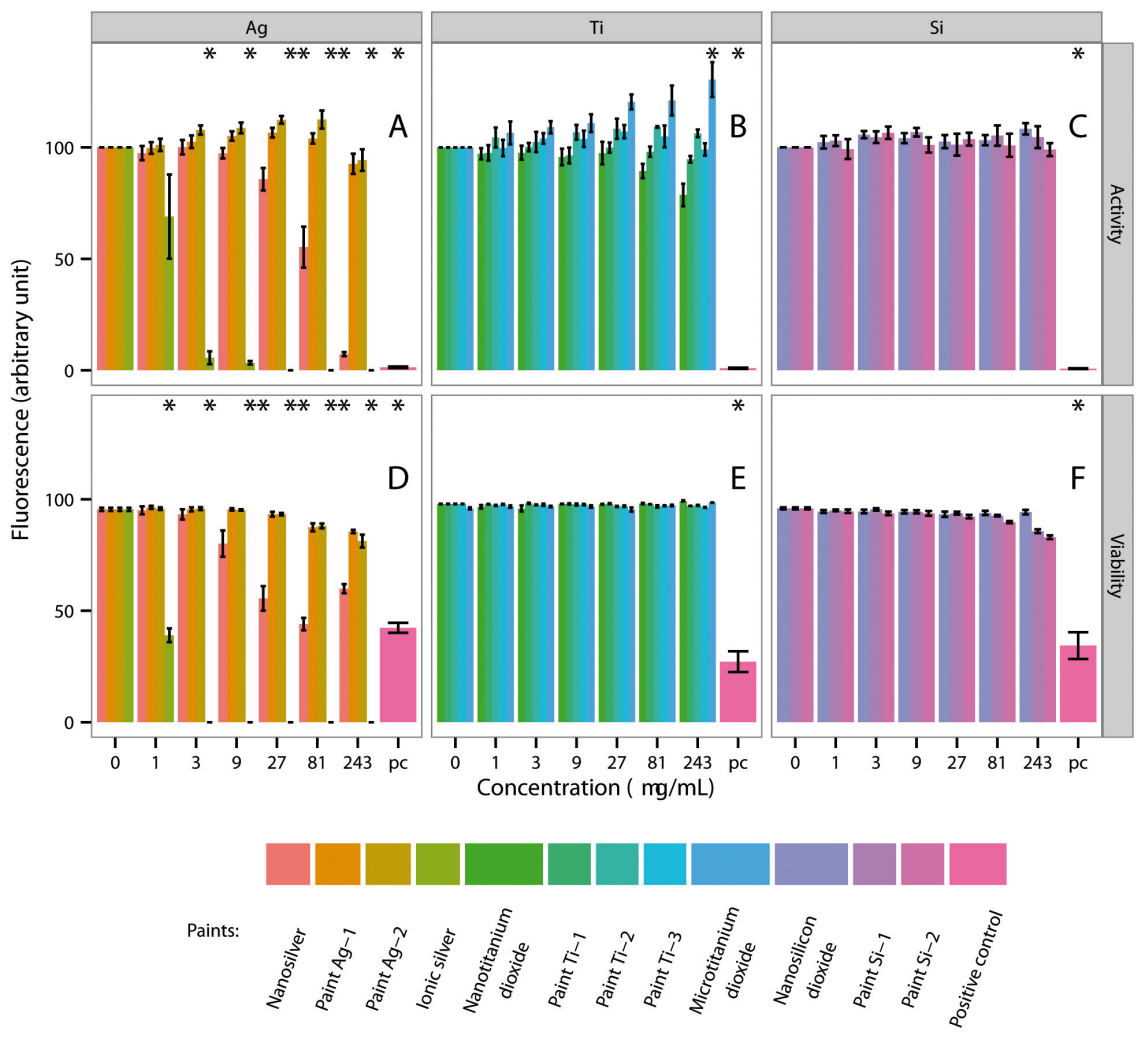
Activity and viability of gastrointestinal tract cells (CaCo-2) after exposure to ENPs, ionic silver, microtitanium dioxide and aged paint particles for 48 h. A = CaCo-2 cells were exposed to different concentrations of nanosilver, paint Ag-1, Ag-2 and ionic silver. B = CaCo-2 cells were exposed to different concentrations of nanotitanium dioxide, paint Ti-1, Ti-2, Ti-3 and microtitanium dioxide. C = CaCo-2 cells were exposed to different concentrations of nanosilicon dioxide, paint Si-1 and Si-2. * = Significantly different from the control.

CaCo-2 cells and Jurkat cells exposed to nano- or microtitanium dioxide, nanosilicon dioxide or the paint particles Ag-1, Ag-2, Ti-1, Ti-2, Ti-3, Si-1 or Si-2 up to a concentration of 243 µg/mL showed no or only a slight reduction in cell activity (Figures 4A-C, S5A-C, S6A-C and S7A-C). Nanosilver was more toxic. A significant reduction in enzymatic activity was observed when CaCo-2 cells were exposed to 81 µg/mL for 48 h. Jurkat cells were more sensitive. A strong reduction in enzymatic activity was observed when these cells were exposed to nanosilver. The effects were more severe when silver was applied in its ionic form. Exposure of CaCo-2 or Jurkat cells to low concentrations of ionic silver resulted in a severe reduction in enzymatic activity (Figures 4A, S5A, S6A and S7A).

After treatment with the three ENPs (nanosilver, nanotitanium dioxide and nanosilicon dioxide) or the paint particles (Ag-1, Ag-2, Ti-1, Ti-2, Ti-3, Si-1 and Si-2), as well as to ionic silver and microtitanium dioxide, cell viability was analysed by live/dead measurements (i.e. apoptosis/necrosis) (Figures 4D-F, S5D-F, S6D-F and S7D-F). No significant effect on cell death was observed when either cell type was exposed to nano- or microtitanium dioxide, nanosilicon dioxide or paint particles Ti-1, Ti-2, Ti-3 in different concentrations (1, 3, 9, 27, 81 and 243 µg/mL) for 24 and 48 h (Figures 4E-F, S5E-F, S6E-F and S7E-F).

Nanosilver was much more toxic. A significant number of necrotic cells were observed after exposure of CaCo-2 cells to 27 µg/mL for 48 h (Figure 4D). Jurkat cells were more robust and significant numbers of dead cells only became apparent on exposure to 81 µg/mL for 48 h (Figure S7D).

Silver applied in its ionic form as silver sulfate severely affected cell behaviour at much lower concentrations. Exposure of CaCo-2 cells to 3 µg/mL ionic silver (4.34 µg/mL Ag_2_SO_4_) for 24 h or to 1 µg/mL (1.45 µg/mL Ag_2_SO_4_) for 48 h caused a significant amount of necrosis. Jurkat cells were more sensitive. A significant number of necrotic Jurkat cells were observed after the cells were exposed to 1 µg/mL ionic silver for 24 or 48 h (Figures 4D, S5D, S6D and S7D).

## Discussion

Painted surfaces are readily colonized by microorganisms because they provide a habitat with all the necessary nutrients. Therefore, most paints contain biocides to prevent microbial colonization. However, biocides or compounds with biocidal effects now have to fulfil the requirements of the *European Biocidal Products Directive 98/8/EC* (BPD); that is, biocides must not accumulate in the environment and must not be ecotoxic or toxic for higher organisms. The paint industry is working on different strategies to create new paint formulations that fulfil the BPD regulations and are able to protect paints from microbial colonization for several years. Thus, the paint industry is not only focusing research on new degradable compounds but is also considering ENPs, which hold great promise for a variety of industrial and consumer applications because of their outstanding and novel properties. ENPs could improve paint characteristics, such as durability, water repellence, scratch resistance, antimicrobial properties, etc, but the fate of these ENPs, once incorporated in the paint matrix, is still very controversial.

The most obvious exposure scenario is during the use and work with ENP-modified paints, when the ENPs could be inhaled, as elaborated by studies using ultrafine particles and ENPs. Particles not cleared by alveolar macrophages or mucociliary clearance may persist in the lungs and induce oxidative stress through increased ROS production. In rare cases, this could lead to chronic inflammation and finally to severe health effects. However, ENPs in paint might also be released during rain events and transferred by run-off water into the surface water and subsequently consumed by animals and/or humans. The safety of these products is not only important for social but also economic reasons.

It is still not proven whether the use of ENP-doped paints is without potential risk. Therefore we investigated potential exposure to ENPs via ingestions and any effects on the immune system in order to enlarge the knowledge of potential cytotoxic effects on tissues other than the lung. We investigated three ENPs (nanosilver, nanotitanium dioxide, nanosilicon dioxide) that have realistic potential to be used by the paint industry to improve paint properties, as well as the corresponding paints with and without these particles, with a special focus on ingestion. The cytotoxic effects of the three ENPs and their corresponding paints were investigated using cells of the gastrointestinal tract (CaCo-2 cells) and of the immune system (Jurkat cells). Because the amount of ENPs that could be released into the environment at any one time is not predictable, we exposed the cells to a broad range of concentrations of the different ENPs, including possible toxic effects at very high exposure (up to 243 µg/mL).

Exposure of CaCo-2 and Jurkat cells to the three selected ENPs or the corresponding paints (Ag-1, Ag-2, Ti-1, Ti-2, Ti-3, Si-1 and Si-2) up to concentrations of 243 µg/mL did not cause morphological changes. However, nanosilver and ionic silver were cytotoxic and cell morphology was affected when cells were exposed to cytotoxic concentrations.

The NanoSight measurements demonstrated that nanosilver and nanotitanium dioxide agglomerated in the culture medium, which affects cellular uptake because, in general, smaller particles are more easily taken up by cells [15,16]. Further, we showed that nanosilver and nanotitanium dioxide were adsorbing peptides/proteins from human blood serum, which would also change the physicochemical properties of the particles and by that their bioavailability and uptake. Nevertheless, TEM demonstrated that nanosilver, nanotitanium dioxide or nanotitanium dioxide containing paint particles (Ti-1, Ti-2, Ti-3) were taken up by CaCo-2 and Jurkat cells. The uptake of paint particles was independent of the presence or absence of nano- or microtitanium dioxide. Nano- or microtitanium dioxide did not influence the incorporation of milled paint particles. Cellular uptake did not lead to changes in cell morphology when the cells were exposed to non-cytotoxic concentrations. Unfortunately, it is still unknown if extended uptake and accumulation of these particles in cells will cause dysfunction after prolonged exposure [17].

ENPs have a high surface area, which often results in higher reactivity and may be relevant for health effects. When CaCo-2 cells or Jurkat cells came in contact with nanosilver, ionic silver, nano- or microtitanium dioxide and nanosilicon dioxide, as well as to paint particles Ag-1, Ag-2, Ti-1, Ti-2, Ti-3, Si-1 and Si-2 (243 µg/mL) for 4 h, no significant amounts of ROS were released. ROS are also able to mediate the activation of transcription factors, which stimulate the release of inflammatory cytokines [18,19]. As expected, the release of cytokines was very low. Only CaCo-2 cells exposed to nanosilver or ionic silver at cytotoxic concentrations released a significant amount of IL-8.

Nanosilver and ionic silver had significant cytotoxicity. Both cell types were severely affected when silver was applied in its ionic form. Exposure to 1 µg/mL ionic silver caused a significant amount of necrosis. Nanosilver was less toxic. A significant number of dead CaCo-2 and Jurkat cells was observed when the cells were exposed to 27 µg/mL and 81 µg/mL nanosilver, respectively. Because of the high chloride concentration in the culture medium, only low amounts of ionic silver were released from the silver nanoparticles. Our data clearly demonstrate that silver nanoparticles are much less cytotoxic than their corresponding metal ions. One of the reasons is that silver nanoparticles are less reactive than silver ions. Silver ions show higher antimicrobial and cytotoxic activity than nanosized silver particles. It has been shown that ionic silver strongly interacts with the thiol-groups of vital enzymes or interacts with DNA [20]. Silver ions also have a tendency to form complexes with various ligands and these silver ligand complexes can be easily transported across the plasma membrane via ligand transport systems [21]. In addition, silver ions may enter cells by proton-coupled sodium ion channels [22].

The results of our *in vitro* studies with cells representing the gastrointestinal tract and immune system are in accordance with those of studies by other researchers who investigated the effects of paint dust *in vivo* and *in vitro*. Saber et al. demonstrated that intratracheal instillation of a single dose of dust from ENP-containing paints in mice did not result in an increased toxic effect compared with dust from a conventional paint without ENPs [23]. Observable effects, such as oxidative stress, inflammation or DNA damage, were independent of the presence of ENPs in the paint. Another study showed there was no additive effect of nanotitanium dioxide in paints. Mice instilled with sanding dust from nanotitanium dioxide-containing paints showed no hepatic histopathological differences, as compared with sanding dust from paint without nanotitanium dioxide [24].

Similar findings are reported for amorphous silicon dioxide. The overall toxicity of amorphous silicon dioxide is low, in contrast to crystalline silicon dioxide (quartz), which causes pulmonary diseases such as silicosis or lung cancer [25]. Several inhalation studies using respirable amorphous silicon dioxide particles demonstrated that the particles produced a time- and dose-related local inflammation response in animals’ lung tissues, although the response was transient and reversible after termination after termination of exposure [26,27]. Similar results were reported from *in vitro* studies with human endothelial cells that were exposed to sanding dust from ENP-containing paints and reference paints without ENPs. Dust from the ENP-containing paints did not lead to higher ROS production and did not have greater cytotoxic effects than dust from the reference paints [28]. Pristine ENPs had a larger effect on a mass basis than sanding dust from paints.

Nanosilver, nanotitanium dioxide and silicon dioxide had been in human use for decades as part of various commercial available products [14,29]. Therefore, it is not unrealistic that the paint manufacturers use these ENPs in order to improve the paint properties. It has already been mentioned before that ENPs attached to paint particles had a lower effect on a mass basis than the ENPs themselves. Regarding the biological effects (health) of different ENPs it has been reported that nanosilver, nanotitanium dioxide and silicon dioxide are compared to combustion derived particles (e.g. carbon black, carbon nanotubes) less likely to cause acute adverse health effects [30–32].

Using an abrasion study, others have compared the release of ENPs from ENP-doped paints with the release of ENPs (including paint particles) from conventional paints without ENPs. Those studies revealed that a smaller number of ENPs and a larger number of micro-sized particles were released [5,33]. The size of the released particles was independent of the ENPs in the paint formulation, but dependent on the hardness of the paint and the grit size of the sanding paper, as well as the pressure applied during the sanding process. Thus, incorporating ENPs into paints will not lead to a severe increase in the number of ENPs in the environment.

There is a concern that ENPs in paints might also be released from painted surfaces during rain events and transferred by run-off water into the surface water. The leaching of microtitanium dioxide from a paint that contained it as pigment was studied by Kaegi et al. [34]. Most of the released pigment was embedded in an organic binder that was part of the paint matrix. In accordance with that finding, we assume that nanotitanium dioxide particles that are incompletely embedded in paint particles will be released during first rain events, will agglomerate and adsorb to other particles and by this process will not persist in the water column, but rather be transferred into sediments where they are less bioavailable and thus not accumulated in the food chain.

## Conclusions

The *in vitro* studies of CaCo-2, Jurkat and endothelial cells exposed to ENPs and to corresponding paint particles with and without these ENPs indicate that there are no significant differences concerning cytotoxicity when cells are exposed to ENP-doped paints, when compared with the same paints without ENPs. Similar findings have been reported for *in vivo* studies using paint dust. The acute toxic effects of ENP-containing paints are similar to those of conventional paints without ENPs. In addition, the cytotoxic effect of the pure ENPs is stronger than the effect of the same particles incorporated in a paint matrix.

The majority of the released ENPs are embedded in the paint matrix and single ENPs will agglomerate and adsorb to other particles on release, meaning accumulation in high amounts in the food chain is unlikely. Thus, the selected ENPs should not pose additional acute health risks. However, studies investigating the long-term impacts of ENP exposure are rare. It is still unknown if the extended incorporation and the accumulation of ENPs in cells will cause dysfunction after prolonged exposure. Therefore predictions concerning chronic health effects after continued ENP uptake are currently not possible. Another subject is that ENPs adsorbed to other particles will be partly transferred into sediments. The ENPs released from paints will lead to an additional accumulation of ENPs in the sediments. Thus, a creeping immission into the environment cannot be excluded and the consequences were not fully assessed yet.

## Supporting Information

Table S1 **Size distribution analysis of nanosilver and nanotitanium dioxide particles.** *TEM, transmission electron microscopy.
(DOCX)

Figure S1 **Behaviour of nanosilver and nanotitanium dioxide in culture medium with 10% fetal calf serum.** A = The measured size of nanosilver agglomerates is in the range of 25–300 nm. Most agglomerates were found in the range of 50–200 nm, with maxima around 125 nm. No visible change in the nanosilver agglomerates was observed during the 48-h incubation period. The nanosilver agglomerates, which were already present at the beginning of the incubation period, did not form larger agglomerates. B = A high amount of non-agglomerated nanotitanium dioxide was observed after dispersing the particles in the culture medium. The amount of non-agglomerated nanotitanium dioxide decreased with increasing incubation time. Nanotitanium dioxide started forming agglomerates of up to 250–300 nm during the 48-h incubation in the culture medium.
(TIF)

Figure S2 **Morphology of gastrointestinal cells (CaCo-2) after exposure to different concentrations of nanotitanium dioxide for 48 h.** A = 1 µg/mL, B = 3 µg/mL, C = 9 µg/mL, D = 27 µg/mL, E = 81 µg/mL, F = 243 µg/mL.
(TIF)

Figure S3 **Uptake of nanosilver, nanotitanium dioxide, and paint Ti-1 particles by immune system cells cells (Jurkat).** A = Jurkat cells grown in the absence of both ENPs and paint particles. B = Jurkat cell culture exposed to nanosilver (27 µg/mL) for 48 h. The uptake of nanosilver by Jurkat cells was much slower than by CaCo-2 cells. 1: Nanosilver agglomerates. C = Jurkat cells exposed to nanotitanium dioxide (243 µg/mL) for 48 h. The uptake of nanotitanium dioxide by Jurkat cells was much slower than by CaCo-2 cells. 2: Nanotitanium dioxide agglomerates. D = Jurkat cell culture exposed to paint Ti-1 particles shows incorporation of the particles. 3: Nanotitanium dioxide agglomerates, 4: Microtitanium dioxide agglomerates, 5: Agglomerates of paint particles. Paint particles, as well as nanotitanium dioxide and microtitanium dioxide particles, were taken up by the cells and incorporated into the cell body without affecting cell behaviour.
(TIF)

Figure S4 **Production of reactive oxygen species (ROS) by immune system cells (Jurkat) after exposure to ENPs, ionic silver, microtitanium dioxide and aged paint particles for 4 h.** A = Jurkat cells were exposed to different concentrations of nanosilver, paint Ag-1, Ag-2 and ionic silver. B = Jurkat cells were exposed to different concentrations of nanotitanium dioxide, paint Ti-1, Ti-2, Ti-3 and microtitanium dioxide. C = Jurkat cells were exposed to different concentrations of nanosilicon dioxide, paint Si-1 and Si-2. * = Significantly different from the control.
(TIF)

Figure S5 **Activity and viability of gastrointestinal tract cells (CaCo-2) after exposure to ENPs, ionic silver, microtitanium dioxide and aged paint particles for 24 h.** A = CaCo-2 cells were exposed to different concentrations of nanosilver, paint Ag-1, Ag-2 and ionic silver. B = CaCo-2 cells were exposed to different concentrations of nanotitanium dioxide, paint Ti-1, Ti-2, Ti-3 and microtitanium dioxide. C = CaCo-2 cells were exposed to different concentrations of nanosilicon dioxide, paint Si-1 and Si-2. * = Significantly different from the control.
(TIF)

Figure S6 **Activity and viability of immune system cells (Jurkat) after exposure to ENPs, ionic silver, microtitanium dioxide and aged paint particles for 24 h.** A = Jurkat cells were exposed to different concentrations of nanosilver, paint Ag-1, Ag-2 and ionic silver. B = Jurkat cells were exposed to different concentrations of nanotitanium dioxide, paint Ti-1, Ti-2, Ti-3 and microtitanium dioxide. C = Jurkat cells were exposed to different concentrations of nanosilicon dioxide, paint Si-1 and Si-2. * = Significantly different from the control.
(TIF)

Figure S7 **Acitivity and viability of immune system cells (Jurkat) after exposure to ENPs, ionic silver, microtitanium dioxide and aged paint particles for 48 h.** A = Jurkat cells were exposed to different concentrations of nanosilver, paint Ag-1, Ag-2 and ionic silver. B = Jurkat cells were exposed to different concentrations of nanotitanium dioxide, paint Ti-1, Ti-2, Ti-3 and microtitanium dioxide. C = Jurkat cells were exposed to different concentrations of nanosilicon dioxide, paint Si-1 and Si-2. * = Significantly different from the control.
(TIF)
