# Supplementary material for: Human Health Risk of Ingested Nanoparticles That Are Added as Multifunctional Agents to Paints: an In Vitro Study
Source: PLoS One. 2013 Dec 16;8(12):e83215. doi: 10.1371/journal.pone.0083215 (PMC3865187; doi:10.1371/journal.pone.0083215)
Supplement: Table S1 — Size distribution analysis of nanosilver and nanotitanium dioxide particles. *TEM, transmission electron microscopy. (DOCX) [file pone.0083215.s001.docx]

**Table S1. Size distribution analysis of nanosilver and nanotitanium dioxide particles**

| **Parameter** | **Nanosilver** | **Nanotitanium dioxide** |
| --- | --- | --- |
| TEM* size (nm) | 25 | 15 |
| Size distribution range (nm) | 25–300 | 5–150 |
| Highest amount of agglomerates/single nanoparticles (nm) | 50–200 | 15 |
| Maxima of agglomerates/single nanoparticles (nm) | 125 | 15 |
