# Supplementary figures and images for: Human Health Risk of Ingested Nanoparticles That Are Added as Multifunctional Agents to Paints: an In Vitro Study
Source: PLoS One. 2013 Dec 16;8(12):e83215. doi: 10.1371/journal.pone.0083215 (PMC3865187; doi:10.1371/journal.pone.0083215)

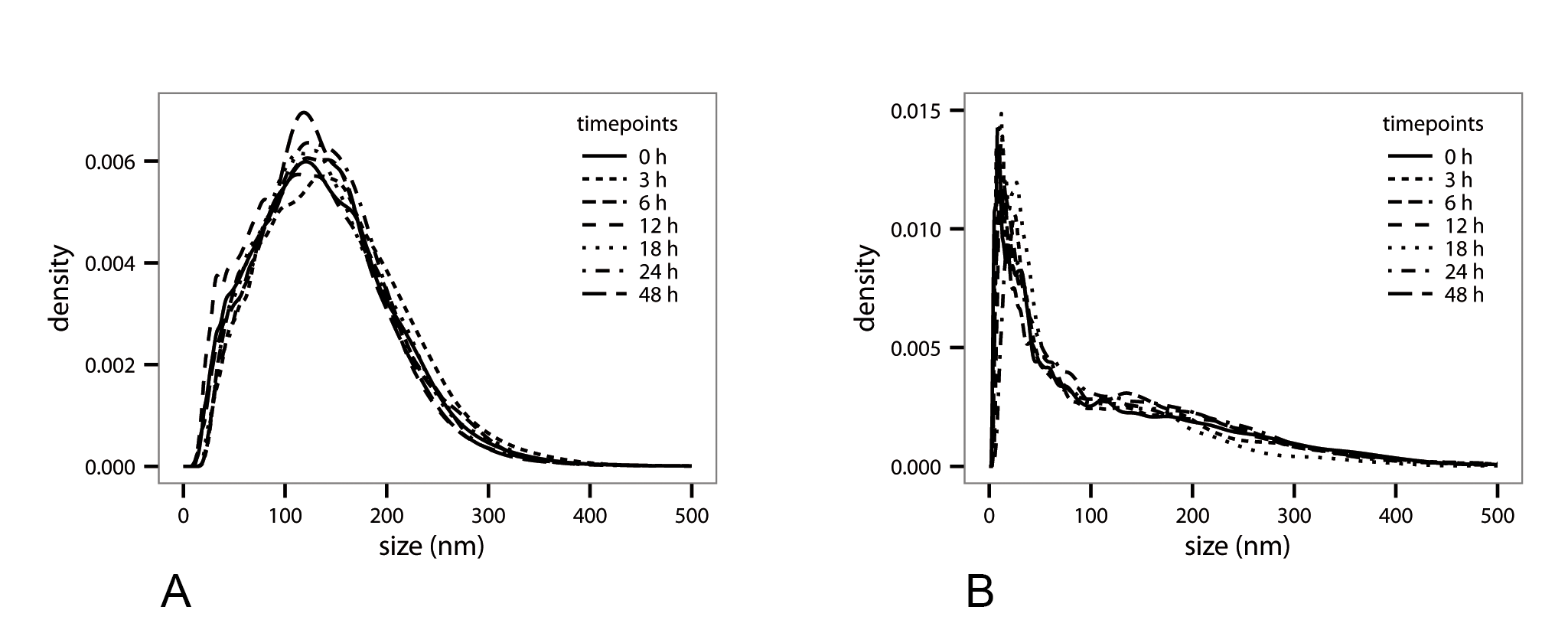

Supplement: Figure S1 — Behaviour of nanosilver and nanotitanium dioxide in culture medium with 10% fetal calf serum. A = The measured size of nanosilver agglomerates is in the range of 25–300 nm. Most agglomerates were found in the range of 50–200 nm, with maxima around 125 nm. No visible change in the nanosilver agglomerates was observed during the 48-h incubation period. The nanosilver agglomerates, which were already present at the beginning of the incubation period, did not form larger agglomerates. B = A high amount of non-agglomerated nanotitanium dioxide was observed after dispersing the particles in the culture medium. The amount of non-agglomerated nanotitanium dioxide decreased with increasing incubation time. Nanotitanium dioxide started forming agglomerates of up to 250–300 nm during the 48-h incubation in the culture medium. (TIF) [file pone.0083215.s002.tif]

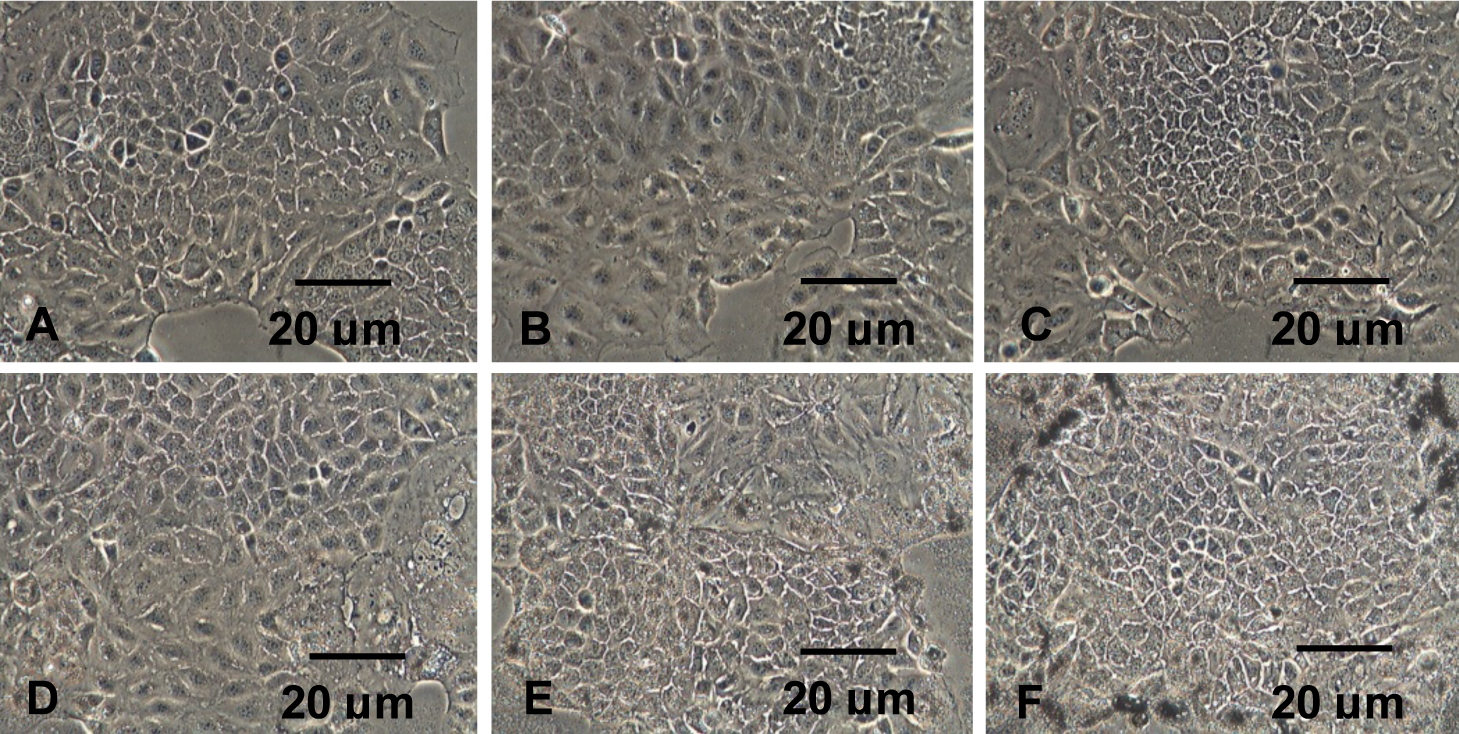

Supplement: Figure S2 — Morphology of gastrointestinal cells (CaCo-2) after exposure to different concentrations of nanotitanium dioxide for 48 h. A = 1 µg/mL, B = 3 µg/mL, C = 9 µg/mL, D = 27 µg/mL, E = 81 µg/mL, F = 243 µg/mL. (TIF) [file pone.0083215.s003.tif]

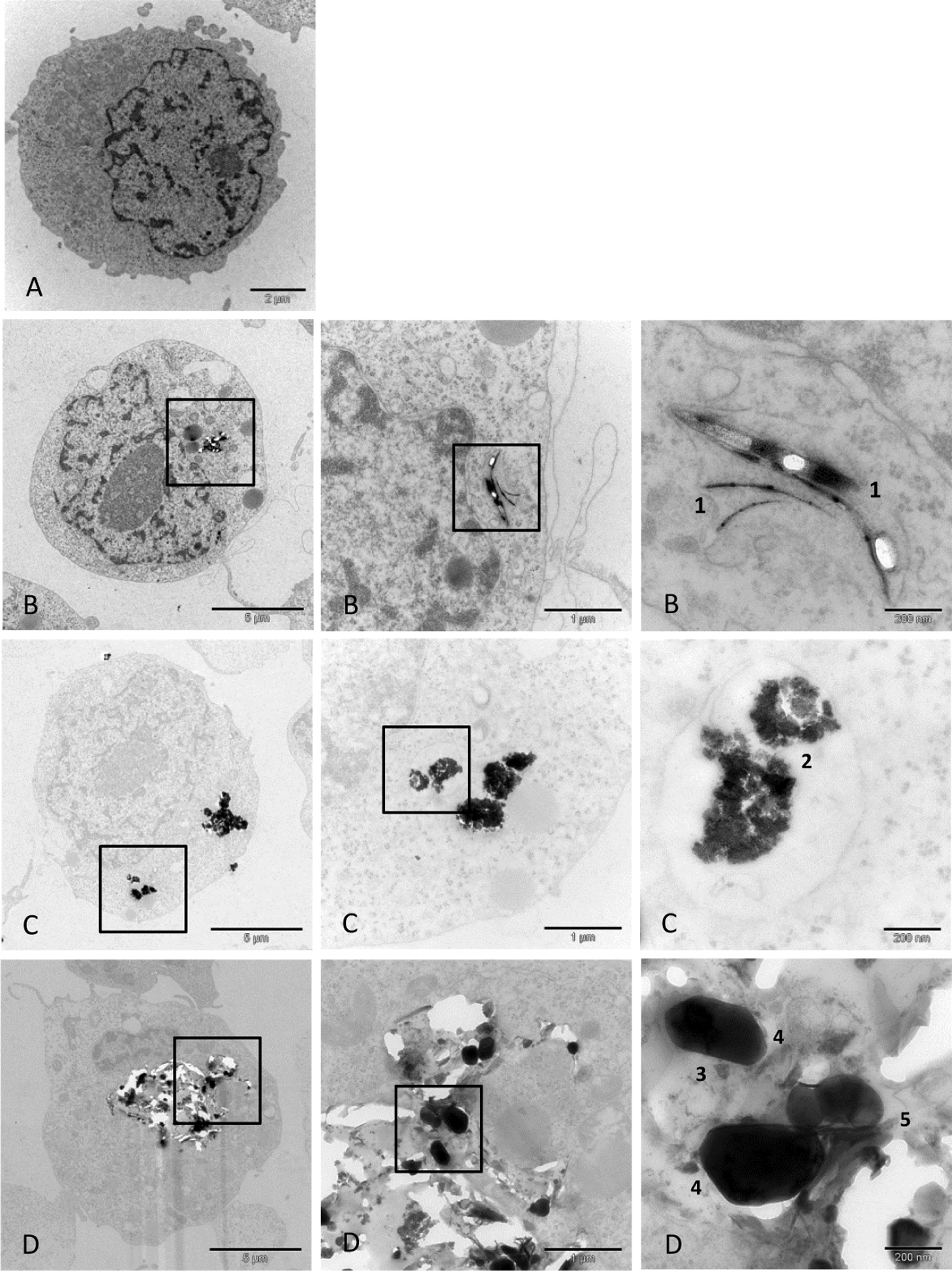

Supplement: Figure S3 — Uptake of nanosilver, nanotitanium dioxide, and paint Ti-1 particles by immune system cells cells (Jurkat). A = Jurkat cells grown in the absence of both ENPs and paint particles. B = Jurkat cell culture exposed to nanosilver (27 µg/mL) for 48 h. The uptake of nanosilver by Jurkat cells was much slower than by CaCo-2 cells. 1: Nanosilver agglomerates. C = Jurkat cells exposed to nanotitanium dioxide (243 µg/mL) for 48 h. The uptake of nanotitanium dioxide by Jurkat cells was much slower than by CaCo-2 cells. 2: Nanotitanium dioxide agglomerates. D = Jurkat cell culture exposed to paint Ti-1 particles shows incorporation of the particles. 3: Nanotitanium dioxide agglomerates, 4: Microtitanium dioxide agglomerates, 5: Agglomerates of paint particles. Paint particles, as well as nanotitanium dioxide and microtitanium dioxide particles, were taken up by the cells and incorporated into the cell body without affecting cell behaviour. (TIF) [file pone.0083215.s004.tif]

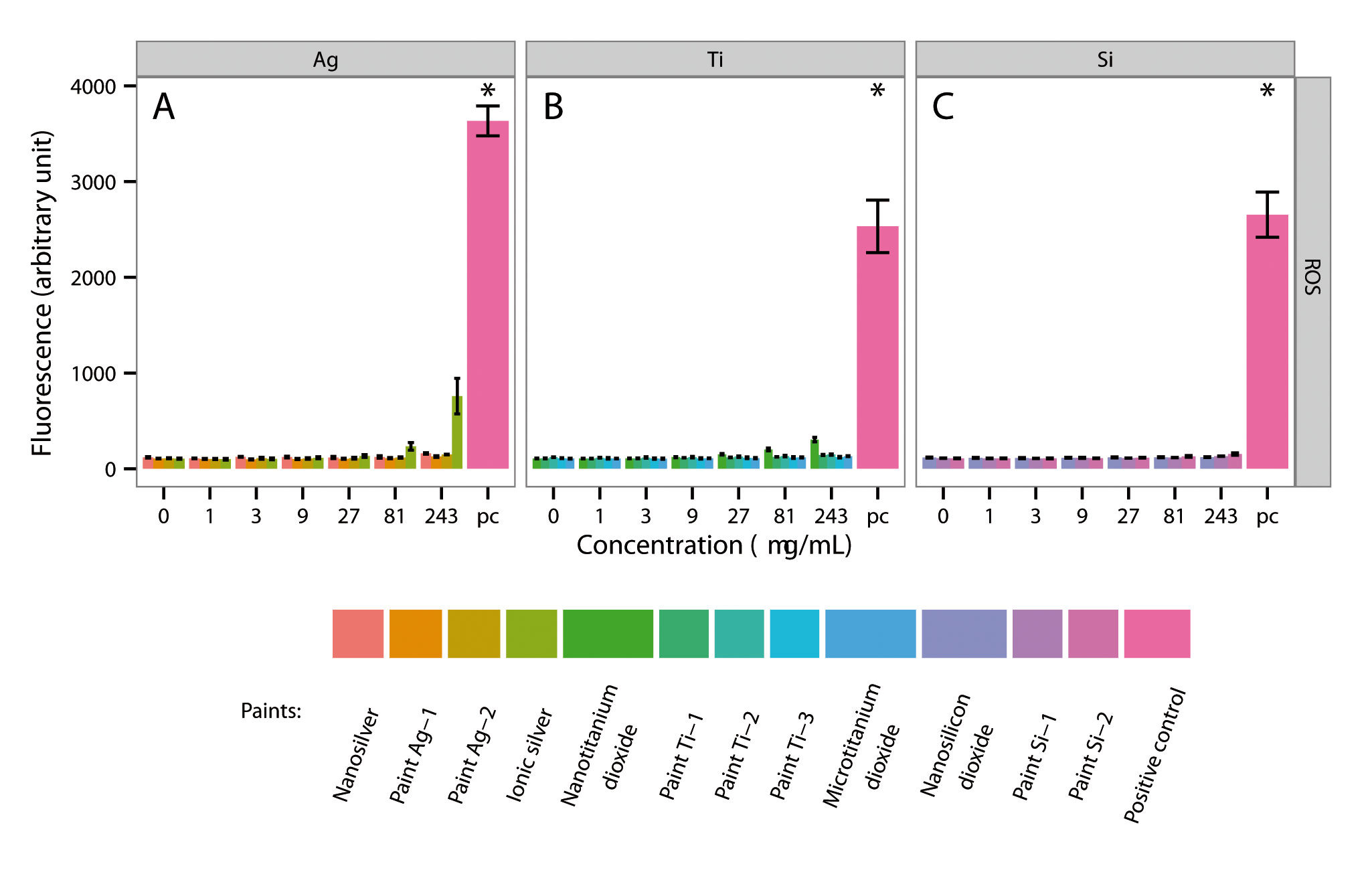

Supplement: Figure S4 — Production of reactive oxygen species (ROS) by immune system cells (Jurkat) after exposure to ENPs, ionic silver, microtitanium dioxide and aged paint particles for 4 h. A = Jurkat cells were exposed to different concentrations of nanosilver, paint Ag-1, Ag-2 and ionic silver. B = Jurkat cells were exposed to different concentrations of nanotitanium dioxide, paint Ti-1, Ti-2, Ti-3 and microtitanium dioxide. C = Jurkat cells were exposed to different concentrations of nanosilicon dioxide, paint Si-1 and Si-2. * = Significantly different from the control. (TIF) [file pone.0083215.s005.tif]

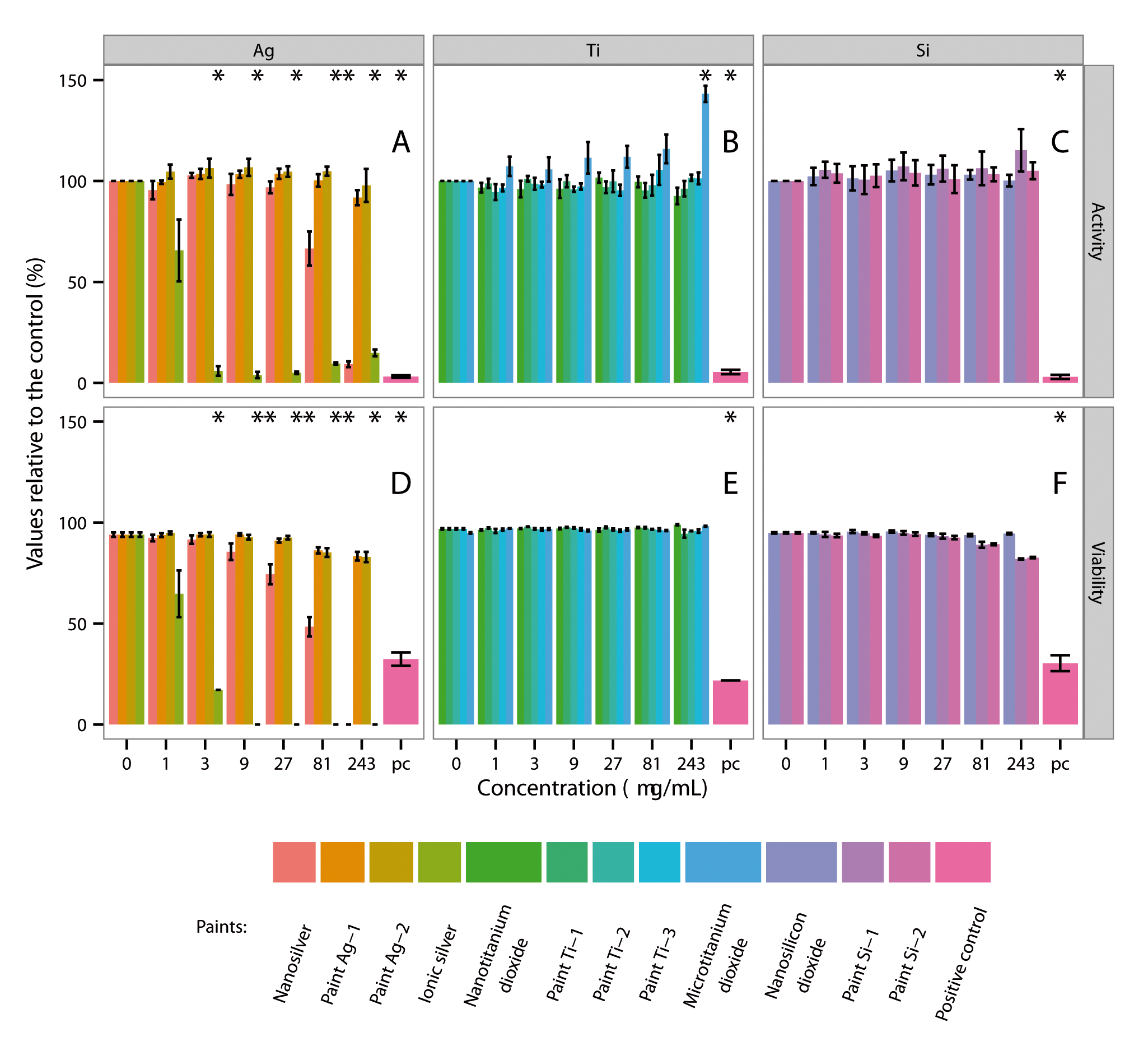

Supplement: Figure S5 — Activity and viability of gastrointestinal tract cells (CaCo-2) after exposure to ENPs, ionic silver, microtitanium dioxide and aged paint particles for 24 h. A = CaCo-2 cells were exposed to different concentrations of nanosilver, paint Ag-1, Ag-2 and ionic silver. B = CaCo-2 cells were exposed to different concentrations of nanotitanium dioxide, paint Ti-1, Ti-2, Ti-3 and microtitanium dioxide. C = CaCo-2 cells were exposed to different concentrations of nanosilicon dioxide, paint Si-1 and Si-2. * = Significantly different from the control. (TIF) [file pone.0083215.s006.tif]

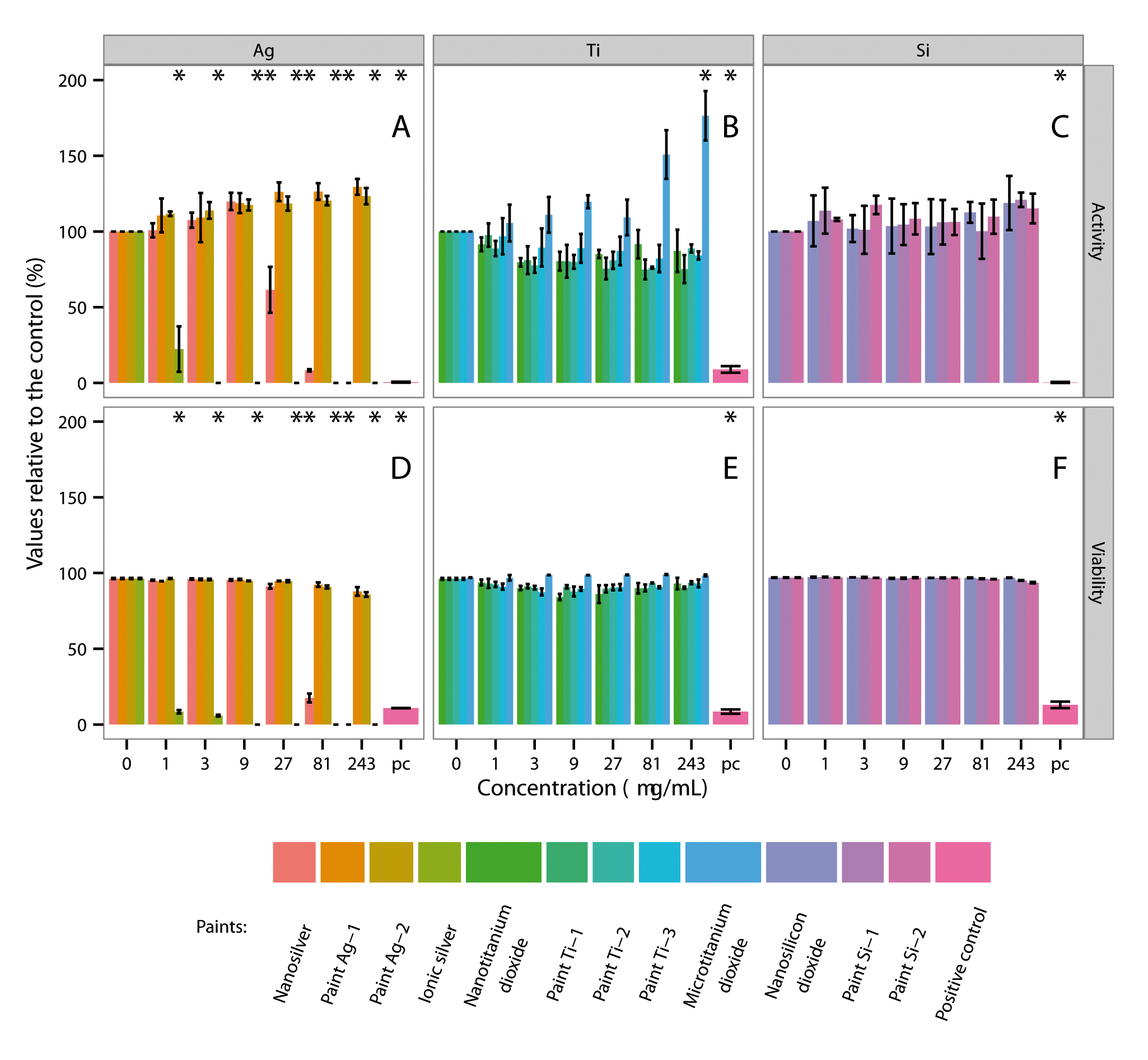

Supplement: Figure S6 — Activity and viability of immune system cells (Jurkat) after exposure to ENPs, ionic silver, microtitanium dioxide and aged paint particles for 24 h. A = Jurkat cells were exposed to different concentrations of nanosilver, paint Ag-1, Ag-2 and ionic silver. B = Jurkat cells were exposed to different concentrations of nanotitanium dioxide, paint Ti-1, Ti-2, Ti-3 and microtitanium dioxide. C = Jurkat cells were exposed to different concentrations of nanosilicon dioxide, paint Si-1 and Si-2. * = Significantly different from the control. (TIF) [file pone.0083215.s007.tif]

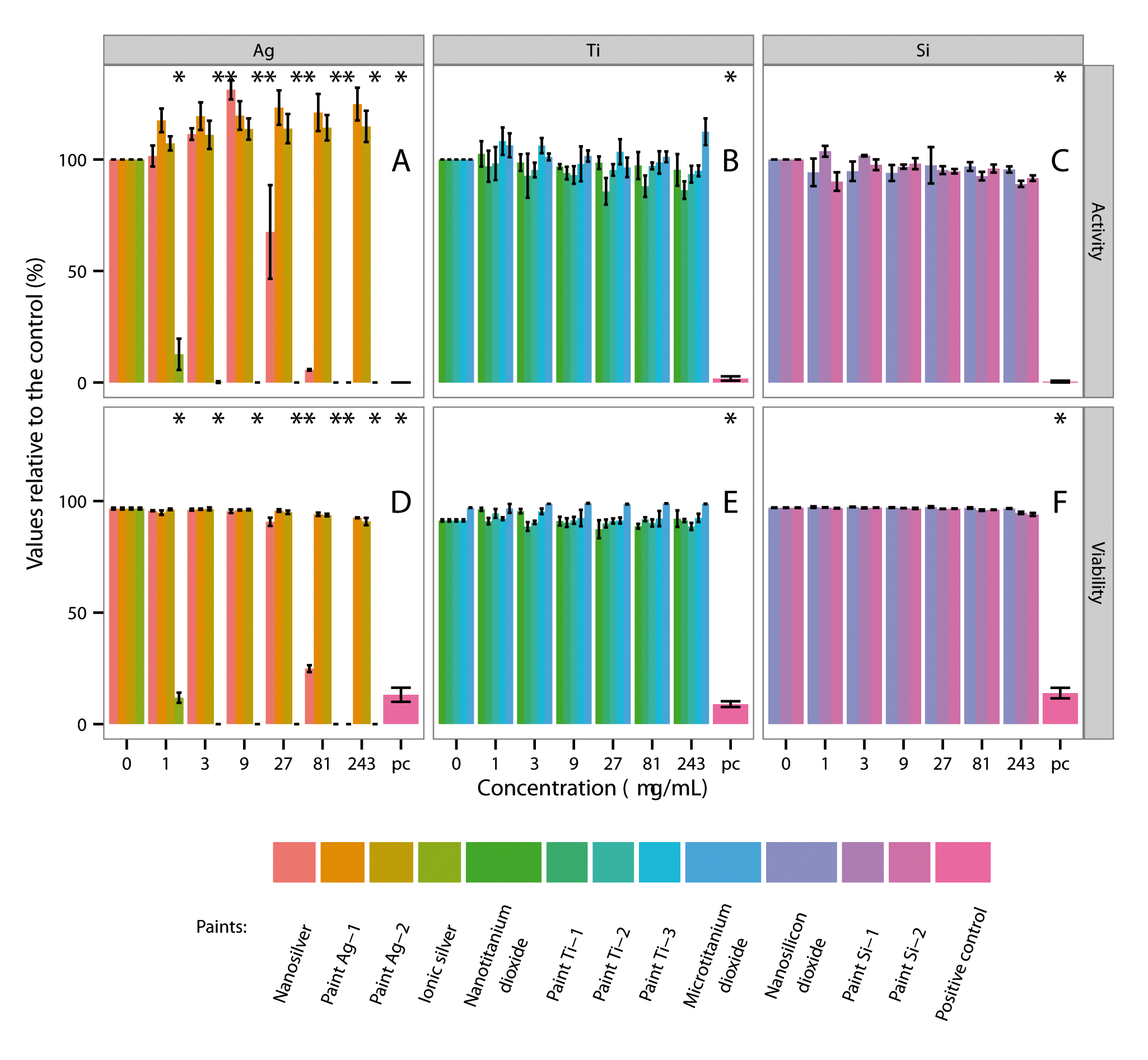

Supplement: Figure S7 — Acitivity and viability of immune system cells (Jurkat) after exposure to ENPs, ionic silver, microtitanium dioxide and aged paint particles for 48 h. A = Jurkat cells were exposed to different concentrations of nanosilver, paint Ag-1, Ag-2 and ionic silver. B = Jurkat cells were exposed to different concentrations of nanotitanium dioxide, paint Ti-1, Ti-2, Ti-3 and microtitanium dioxide. C = Jurkat cells were exposed to different concentrations of nanosilicon dioxide, paint Si-1 and Si-2. * = Significantly different from the control. (TIF) [file pone.0083215.s008.tif]
